# Rehabilitation Outcomes in Subjects with West Nile Neuro-Invasive Disease

**DOI:** 10.3390/brainsci11101253

**Published:** 2021-09-22

**Authors:** Komal Patel, Brian D. Greenwald, Rosanna C. Sabini

**Affiliations:** 1 Division of Physical Medicine and Rehabilitation, The George Washington University Medical Faculty Associates, Washington, DC 20037, USA; 2 JFK Johnson Rehabilitation Center for Brain Injuries, Hackensack-Meridian School of Medicine, Edison, NJ 08818, USA; brian.greenwald@hmhn.org; 3 Department of Physical Medicine & Rehabilitation, Donald and Barbara Zucker School of Medicine at Hofstra/Northwell Health, South Shore University Hospital, Bay Shore, NY 11706, USA; rsabini@northwell.edu

**Keywords:** West Nile Neuro-invasive Disease, West Nile Virus, acute inpatient brain injury rehabilitation, outcomes

## Abstract

West Nile Virus (WNV) is the most common mosquito borne cause of viral encephalitis in the United States. Physical and neuro-cognitive recovery from WNND may be prolonged or incomplete leading to chronic cognitive inefficiencies and functional decline. There continues to be no effective treatment of WNV and current management is primarily supportive. The objective of this review is to evaluate the functional outcomes and role of rehabilitation services in subjects with WNND. The charts of five subjects admitted to an acute inpatient brain injury rehabilitation facility from June to December 2012 were retrospectively reviewed. (Mean, Range)-Age (64.8, 43–78 years), Admission Functional Independence Measure (FIM) (45.2, 14–63), Discharge FIM (82.2, 61–100), FIM score gain (37, 24–60), Cognitive FIM gain (7, 1–18), Mobility FIM gain (17.4, 13–20), ADL FIM gain (12.6, 4–23); acute brain injury inpatient rehabilitation facility length of stay (LOS) (17.8, 14–21 days); acute hospital LOS (15, 10–22 days). Of the five subjects, three were discharged home, one was discharged to a skilled nursing facility, and one was discharged to an assisted living facility. Subjects with WNND have significant functional decline across all FIM subcategories and may benefit from a course of brain injury-specific acute inpatient rehabilitation.

## 1. Introduction

From 1999 to 2019, the Centers for Disease Control and Prevention (CDC) reported over 51,000 cases of West Nile Virus (WNV) throughout the United States [1]. During that same time period, the CDC also reported over 25,000 cases that classified as West Nile Neuro-invasive Disease (WNND), approximately 49% of cases [1]. WNV is now the most common mosquito borne cause of viral encephalitis in the United States [2].

West Nile Virus is an arbovirus transmitted via a mosquito vector that is believed to travel via infected migratory birds [3,4,5,6]. Infection in humans can range from generalized illnesses with fevers, muscle aches, rash, and lymphadenopathy, to a more severe disease causing physical and cognitive impairments [4]. West Nile Neuro-invasive Disease can manifest as meningitis, encephalitis or myelitis and can lead to a reduced level of consciousness, parkinsonian movement disorders, seizures, and/or flaccid paralysis [3]. Factors that determine who is at risk for neuro-invasive disease remain unclear; however, individuals with advanced age are known to have greater severity of WNND [3,6,7]. Despite many of these individuals living independently prior to contracting the viral infection, physical and neuro-cognitive recovery from WNND may be prolonged or incomplete leading to chronic cognitive inefficiencies and functional decline in mobility and activities of daily living (ADLs).

West Nile Virus continues to be a global health dilemma, causing a potentially devastating and life-altering illness, especially when considering the neuro-invasive counterpart. To date, there continues to be no proven effective treatment of WNV [3,4]. Current management for WNV is primarily supportive, although many available agents are under clinical investigation, including interferon, ribavirin, and intravenous immunoglobulin [3,4,8]. With such limited treatment options, severe disability and death can be a result [8]. In a clinical report of 19 subjects hospitalized for WNV, only 7 subjects recovered fully. An additional 10 subjects recovered, but not to their previous functional levels, and 2 of the subjects had died [9].

As with other brain injuries, given the neuro-invasiveness of WNV, acute inpatient brain injury rehabilitation could be considered an option to improve functional deficits in affected individuals. This retrospective review sought to determine if subjects diagnosed with WNNV would benefit from a course of acute inpatient brain injury rehabilitation and improve functional recovery for reintegration into the community.

## 2. Materials and Methods

A retrospective chart review was performed with the approval of the local Institutional Review Board and in accordance with the Declaration of Helsinki. Between June to December 2012, there was an outbreak of WNV in the greater New York area. Subjects diagnosed with West Nile Neuro-invasive Disease who were admitted to a single acute inpatient brain injury rehabilitation facility between June and December 2012 were included in this review. Diagnosis of WNV was confirmed during their acute hospital course via serology or cerebrospinal fluid analysis.

Data extracted from chart review included subject demographics, including age, gender, past medical history and prior functional status. In addition, radiographic imaging, serology and laboratory results were obtained. Length of stay for acute care hospitalization and acute inpatient brain injury rehabilitation was also documented. The acute inpatient brain injury rehabilitation program consisted of a total of 3 h of combined physical, occupational, and speech therapies per day, five days a week. Each subject received an evaluation from all three rehabilitation disciplines to determine the admission Functional Independence Measure (FIM) score. Each subject was then reevaluated at the end of their rehabilitation course to determine their discharge FIM score. Each subject’s discharge location was also noted.

**Figure 1:**
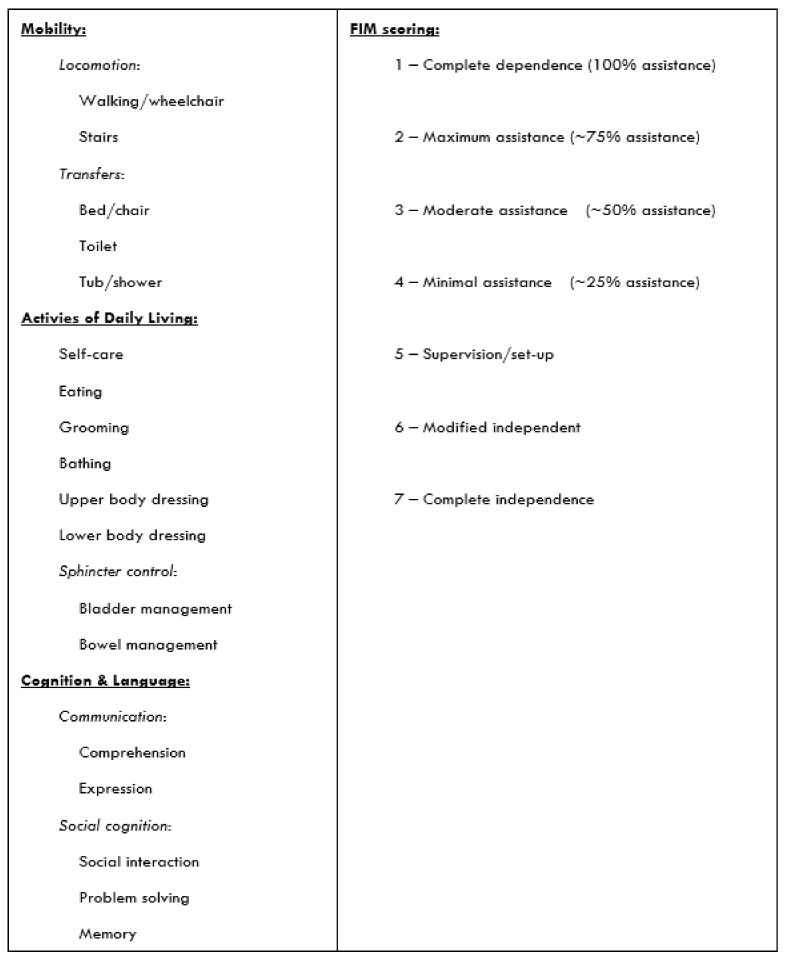
Functional Independence Measure.

The FIM was used to objectively measure subject’s functional recovery, as it is a well-validated and widely utilized tool for assessing functional ability and the need for assistance at the beginning, during, and end of the subject’s rehabilitation course [10,11]. The total FIM score comprises 18 functional items spanning 6 domains, including self-care, locomotion, transfers, communication, sphincter control, and social cognition [10,11]. Each functional item is scored on a scale from 1 to 7, where a 1 is defined as complete dependence and a 7 is defined as complete independence. In Figure 1, a lower total score represents the need for a higher level of assistance. For this study, the 18 functional items were divided into three subcategories that represent mobility, ADL function, and speech, language and cognition.

## 3. Results

A total of five subjects met the inclusion criteria for this retrospective chart review. No subjects admitted during this time frame with WNND were excluded from the study. The study sample comprised three males and two females, with a mean age of 64.8 years. Prior to hospitalization, all subjects were functionally independent in ambulation, transfers, activities of daily living, and cognition, and were also driving (Table 1).

The average length of stay for the acute care hospitalization ranged from 10 to 22 days with a mean of 15 days (Table 2).

Serological testing provided confirmation of West Nile Virus infection in subject 2 and 3. Neither of these subjects had lumbar punctures completed for objective confirmation of West Nile Neuro-invasive Disease; however, both subjects showed symptoms that lucidly depicted neurological involvement of their infection. Subject 2 showed symptoms of aphasia, nystagmus, and right upper extremity weakness. Subject 3 was treated after a motor vehicle accident that was a result of altered mental status and weakness. A lumbar puncture provided confirmation of West Nile Neuro-invasive Disease infection for subjects 1, 4, and 5. Subject 1 showed symptoms of diplopia, nausea, dizziness, and altered mental status. Subject 4 showed symptoms of generalized weakness, nausea, vomiting, fevers, and altered mental status. Subject 5 showed symptoms of altered mental status, ataxia, and diplopia (Table 3).

During this time, each subject underwent brain neuroimaging via computerized tomography (CT) and magnetic resonance (MR). All five subjects had no acute findings on head CT. Only two of the five subjects resulted in a positive brain MR finding: one showed mild bilateral subcortical white matter occipital lobe hyperintensities and the other showed bilateral thalamic, peri-aqueduct gray matter, dorsal pons, cerebellar peduncle, and dentate nuclei plaques with no acute demyelination. Once deemed medically optimized from the referring facility, each subject was then discharged to a single acute inpatient brain injury rehabilitation program.

**Figure 2:**
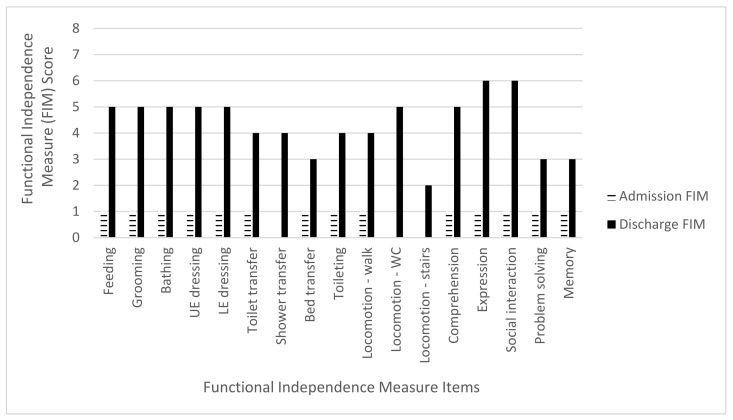
Subject 1 admission and discharge functional independence measure scores.

**Figure 3:**
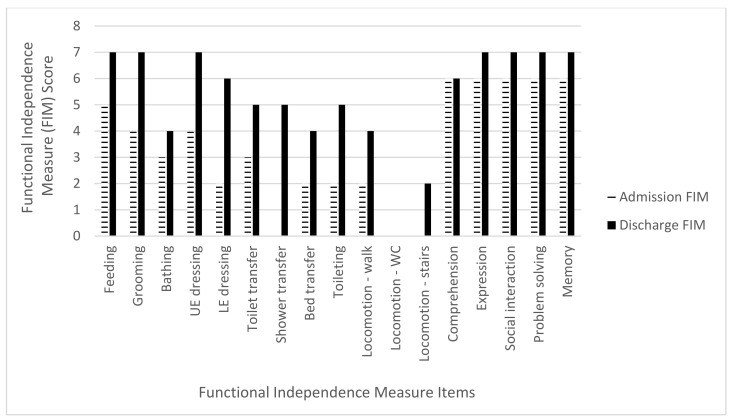
Subject 2 admission and discharge functional independence measure scores.

**Figure 4:**
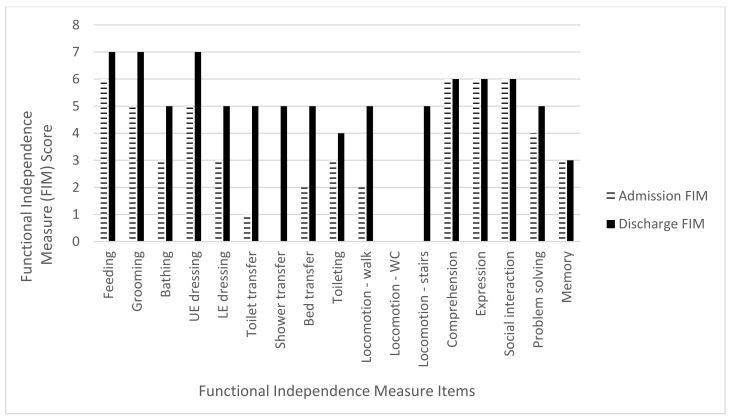
Subject 3 admission and discharge functional independence measure scores.

**Figure 5:**
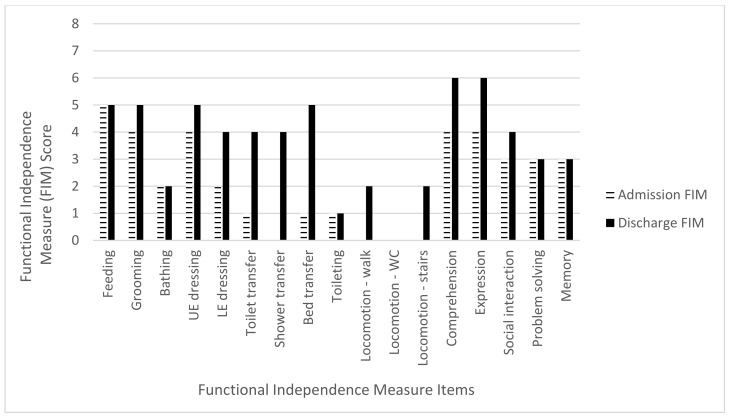
Subject 4 admission and discharge functional independence measure scores.

**Figure 6:**
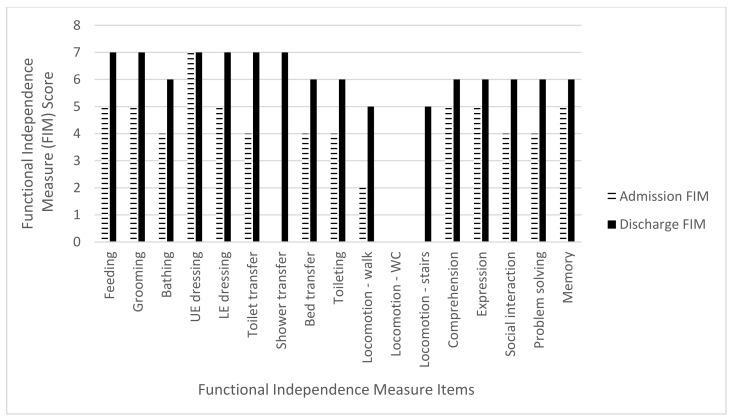
Subject 5 admission and discharge functional independence measure scores.

The mean admission FIM score was 45.2 points (range 14–63 points) while the mean discharge FIM score was 82.2 points (range 61–100). The mean total FIM gain was 37 points (range 24–60 points) with an improvement in all three FIM subcategories. For the mobility subcategory, there was a mean FIM gain of 17.4 points (range 13–20) out of a maximum of 21 points, an 82.8% increase. The ADL subcategory mean FIM gain was 12.6 points (range 4–23) out of a maximum of 63 points, a 20% increase. The cognitive subcategory mean FIM gain was seven points (range 1–18 points) out of a total 35 points, a 20% increase (Table 4 and Figure 2, Figure 3, Figure 4, Figure 5 and Figure 6).

After the subjects achieved the functional goals set forth with the rehabilitation staff during admission, the subjects were referred to the next phase of their rehabilitation course, whether to community living or to a skilled nursing facility. The average length of the acute inpatient brain injury rehabilitation stay was 17.8 days (range of 14–21 days). Four of the five subjects were discharged to the community, and one was discharged to a skilled nursing facility. Of the four subjects discharged to the community, three subjects were discharged to their homes and one subject was discharged to an assisted living facility (Table 1).

## 4. Discussion

Acute inpatient rehabilitation facilities are unique entities that not only provide intensive rehabilitation services, but also the medical oversight needed for more medically complex subjects. Such facilities are mandated by law to maintain a minimum percentage of subjects from specific diagnoses, including brain injury, and provide an intense program of combined 3 h of physical, occupational, and speech therapies per day, five days a week.

West Nile Neuro-invasive Disease qualifies as a brain injury, as it can manifest as meningitis or encephalitis, but a large number of these subjects do not get referred to acute inpatient brain injury rehabilitation facilities. This retrospective review of five subjects diagnosed with WNND demonstrated that these subjects were not only able to tolerate the intensity of an acute inpatient brain injury rehabilitation program but also benefit from it, as shown by their improved Functional Independence Measure scores. Not only did the overall FIM average improve in all subjects, but this review also demonstrated an improvement in all three FIM subcategories for mobility, ADL and cognition. This improvement may have resulted from the more intensive and comprehensive rehabilitation program at an acute inpatient brain injury rehabilitation facility.

This review demonstrated that prior to acute rehabilitation, all subjects had impairments with ambulation, balance, endurance, and stair negotiation, and all were at risk of falls. Mobility plays a large role in assessing community re-integration and the level of assistance needed upon discharge, as well as decreasing caretaker burden. In this review, our subjects improved by an average of 82.8% in the mobility subcategory. This relative improvement made activities such as getting out of bed, going to the bathroom, and stair negotiation easier for subjects and their caretakers to accomplish. In addition to improved mobility, this review also demonstrated that subjects required less assistance in performing ADL tasks, such showering, feeding, grooming, and dressing.

When caring for a subject with a brain injury, discharge planning is oftentimes complicated because of a subject’s cognitive impairments. All five subjects in this review were found to have cognitive inefficiencies and related impairments, which ranged from deficiencies in orientation, simple and divided attention, immediate and sustained memory, restlessness and agitation, to sleep disorders. A course of acute inpatient brain injury rehabilitation for these subjects also showed itself to be beneficial within the cognition subcategory of the FIM.

As demonstrated, all five subjects demonstrated overall improvements in their FIM score over the course of their acute inpatient brain injury rehabilitation stay, of which four subjects were discharged to a community setting. Although this retrospective review noted improvements in functional independence measure scores in subjects with WNND, the sample size was small. A previous case series with the same sample size was also able to show promising results [12]. Despite the small sample sizes, both of these reviews demonstrate promising findings in the setting of a neuro-invasive disease that currently has no proven medical treatment. Therefore, providing subjects with the opportunity to regain functional independence via a course of acute inpatient brain injury rehabilitation may prove to be of benefit.

Looking to the future, studies that include a larger sample size would be beneficial in concluding upon a confirmatory statement. In addition, it may also be of value to follow the functional recovery of subjects diagnosed with WNND from the start of their admission to the acute care hospitalization. Other measures of functional outcomes to consider in future studies are the disability rating scale, the Extended Glasgow Outcome Scale, and return to work, as well as obtaining additional cognitive and ambulation assessments. In this review, only those who had functional impairments and were referred to an acute inpatient brain injury rehabilitation program were included. It is possible that there were other subjects diagnosed with WNND who did not meet the criteria for acute brain injury inpatient rehabilitation after their acute hospitalization, but rather met the criteria for home or a skilled nursing facility discharge or possibly passed away.

## 5. Conclusions

West Nile Neuro-invasive Disease can have a significant impact on overall function and lead to limitations that impart decline in mobility, activities of daily living, and cognition. Despite a small sample size, this retrospective review of subjects with WNND demonstrated that a course of acute inpatient brain injury rehabilitation can improve both physical and cognitive function.

## Figures and Tables

**Table 1:** Subject demographics, co-morbidities, and prior functional abilities.

Subject	Sex	Age (Years)	Co-Morbidities	Prior Ambulation Status	Prior Activities of Daily Living Status	Prior Cognitive Status
1	Male	75	Hypertension, hyperlipidemia, hypothyroidism, osteoarthritis	Independent	Independent	Independent
2	Female	53	Hypertension, seizure disorder, anxiety, cervical disc disease, insomnia	Independent	Independent	Independent
3	Male	78	Coronary artery disease, atrial fibrillation, mitral regurgitation, pulmonary hypertension, dementia, insomnia, enlarged prostate	Independent	Independent	Independent
4	Female	75	Hypertension, hyperlipidemia, diabetes type ii, trigeminal neuralgia, anxiety	Independent	Independent	Independent
5	Male	43	Multiple sclerosis, hyperlipidemia	Independent	Independent	Independent

**Table 2:** Length of stay and disposition location.

Subject	Acute Hospital Length of Stay (LOS)	Acute Inpatient Brain Injury Rehabilitation Facility LOS	Discharge Location
1	12 days	18 days	Home
2	10 days	14 days	Home
3	12 days	19 days	Assisted living
4	19 days	21 days	Skilled nursing facility
5	22 days	17 days	Home

**Table 3:** Subject presenting symptoms, West Nile Virus testing, and imaging studies.

Subject	Presenting Symptoms	Serology	Lumbar Puncture	Head Computed Tomography	Brain Magnetic Resonance Imaging
1	Diplopia, nausea, dizziness, and altered mental status	NA	(+) West Nile	No acute pathology	No acute pathology
2	Aphasia, nystagmus, and right upper extremity weakness	(+) West Nile	NT	No acute pathology	No acute pathology
3	Altered mental status and weakness	(+) West Nile	NT	No acute pathology	No acute pathology
4	Generalized weakness, nausea, vomiting, fevers, and altered mental status	NA	(+) West Nile	No acute pathology	Mild bilateral subcortical white matter occipital lobe hyperintensities
5	Altered mental status, ataxia, and diplopia	NA	(+) West Nile	No acute pathology	Bilateral thalamic, peri-aqueduct, dorsal pons, cerebellar, and dentate nuclei plaques; no acute demyelination

NA = Not available; NT = Not tested.

**Table 4:** Functional independence scores, FIM gain, FIM efficiency.

Subject	Admission FIM	Discharge FIM	FIM Gain	FIM Efficieny
1	14	74	60	3.33
2	57	90	33	2.36
3	55	86	31	1.63
4	37	61	24	1.14
5	63	100	37	2.18

## Data Availability

The data presented in this study are available on request from the corresponding author.
